# Round the Hospitals

**Published:** 1916-11-18

**Authors:** 


**MATRONS' AND SISTERS' DEPARTMENT—(*continued*).**

### ROUND THE HOSPITALS.

Miss G. Cowlin has been appointed assistant secretary of the College of Nursing. She holds the certificate of St. Cross Hospital, Rugby, of St. Bartholomew's Hospital, London, and of Teacher's College, Columbia University, New York, where she studied hospital economics and training-school curricula, a course which, it will be remembered, was also taken by Miss Rundle, the secretary of the College. Miss Cowlin has held the post of home sister at the Queen's Hospital for Children, and of staff nurse, office sister, and assistant matron at No. 1 General Hospital, Camberwell.

Nurses who chafe at the delay in the acceptance or otherwise by the College of Nursing of their applications do not realise the many contributory causes. The difficulty of obtaining adequate clerical assistance will, it is hoped, no longer be one since the appointment of Miss Cowlin as assistant secretary. But a frequent one is the negligence of those whose names have been given as references to answer promptly the inquiries made by the College. In writing to ask permission to give a friend's or employer's name as reference it would be as well for the nurse to include a request that the inquiries which will follow may be answered if possible by return of post. Where this is done the application to become a member can be readily and promptly dealt with and disposed of.

Two meetings in the North, to explain the College of Nursing and its Registration Bill, have been arranged. The first will take place at the Liverpool Royal Infirmary on Friday, November 24, and the second at the Royal Victoria Infirmary, Newcastle-on-Tyne, on Friday, December 8. Both will be addressed by the Hon. Arthur Stanley, who has been indefatigable in addressing meetings up and down the country. There is no more useful way of clearing the ground than for matrons to arrange such meetings, and the Secretary of the College of Nursing will be only too glad to co-operate if she is communicated with at 6 Vere Street, Cavendish Square, London, W. Unfortunately, though the letter-box at 6 Vere Street might appear to belie the supposition, there are very many nurses who would seem to consider themselves but remotely touched by the College of Nursing or registration. It is astonishing that this should be so, and the most effective way of combating this lack of interest and supineness is by meetings, where questions can be asked and answered and the important bearings on the future of nurses and their work which underlie the scheme of the College can be clearly demonstrated.

The Supply of Nurses Committee is proceeding with the collection of statistics regarding the number of nurses trained or in training, but it will be some time before any report can be made. Meantime some changes in the *personnel* of the Committee have been made; Sir Frederick Treves has resigned and two new members have been appointed—viz., Fleet-Surgeon R. W. G. Stewart, R.N., nominated by the Admiralty, and Miss A. MacDonnell, R.R.C., representing the Irish Nurses' Association.

The new Manchester Institute of Massage held its first meeting last week. According to a circular letter issued in October, it was intended that the first Council should be nominated at this meeting, but it was explained that matters had not yet advanced so far as to permit of the appointment of a full Council, and the meeting was adjourned *sine die* after appointing the following officers:—Sir William Cobbett, chairman of the board of management of the Manchester Royal Infirmary, as president; Sir William Milligan, chairman of Council; Dr. A. E. Barclay, honorary secretary; Mr. Jas. L. Fox, honorary treasurer; Messrs. E. Guthrie and Co., auditors. It is intended that the Council shall consist of representatives of hospitals of the United Kingdom, the medical profession, trained workers of both sexes, and members of a university or other educational body. The Institute undertakes to provide centres in all large towns where candidates are being prepared for its examinations. It is stated that the first examination is to be held in January 1917.

Some further research by Miss Stopes at the Foreign Office and the War Office has unearthed the original letter by Miss Nightingale from the Crimea already mentioned in these columns (The Hospital, June 3, 1916, p. 222), on which some high official had made certain uncomplimentary marginal remarks. It turns out that the comment in question runs thus: "I confess I think it is time that we curbed the pretensions of Miss Nightingale to unlimited and almost irresponsible command over the nurses attached to the Army in the East." In her former communication Miss Stopes quoted the tenth word of this passage as "crushed"; but she is now satisfied that it is really "curbed," which certainly makes the minute less offensive, though it does not alter one's opinion of the wrongheadedness of the official, who can be traced, Miss Stopes thinks, in spite of his anonymity. In the course of this research Miss Stopes has come across many other interesting official letters of Miss Nightingale's. Thus, in one from Scutari in December 1914 to the British Embassy at Constantinople, she asks for immediate help in the repair of certain dilapidated hospital buildings. As the Turkish labourers had not been paid, they struck; so Miss Nightingale paid their wages out of her own pocket, and then wrote to the Embassy to know if she had done right. It is pleasant to be able to add that she got a favourable reply, and that the extra accommodation which she wanted for the wounded was forthcoming.

As we go to press we regret to learn that Miss Haughton still remains seriously ill. Though she had a comfortable night her condition is practically unchanged.

**For Matrons' and Sisters' Appointments, see p. 152; for Insurance against Fire of Matrons' and Nurses' Property, see p. 151.**

